# Fatal Meningococcal Disease in a Laboratory Worker — California, 2012

**Published:** 2014-09-05

**Authors:** Channing D. Sheets, Kathleen Harriman, Jennifer Zipprich, Janice K. Louie, William S. Probert, Michael Horowitz, Janice C. Prudhomme, Deborah Gold, Leonard Mayer

**Affiliations:** 1 California Department of Public Health, National Center for Immunization and Respiratory Diseases, CDC; 2 California Division of Occupational Safety and Health, National Center for Immunization and Respiratory Diseases, CDC; 3 Division of Bacterial Diseases, National Center for Immunization and Respiratory Diseases, CDC

Occupationally acquired meningococcal disease is rare (1). Adherence to recommendations for safe handling of *Neisseria meningitidis* in the laboratory greatly reduces the risk for transmission to laboratory workers (2). A California microbiologist developed fatal serogroup B meningococcal disease after working with *N. meningitidis* patient isolates in a research laboratory (laboratory A). The California Department of Public Health (CDPH), the local health department, the California Division of Occupational Safety and Health (CalOSHA), and the federal Occupational Safety and Health Administration (OSHA) collaborated on an investigation of laboratory A, which revealed several breaches in recommended laboratory practice for safe handling of *N. meningitidis*, including manipulating cultures on the bench top. Additionally, laboratory workers had not been offered meningococcal vaccine in accordance with Advisory Committee on Immunization Practices (ACIP) recommendations and CalOSHA Aerosol Transmissible Diseases Standard requirements (3,4). In accordance with OSHA and CalOSHA regulations, laboratory staff members must receive laboratory biosafety training and use appropriate personal protective equipment, and those who routinely work with *N. meningitidis* isolates should receive meningococcal vaccine.

## Case Report

On the evening of Friday, April 27, 2012, a microbiologist aged 25 years had onset of headache, fever, neck pain, and stiffness. The following morning, April 28, he was transported by automobile to the emergency department at hospital A, where he was employed in laboratory A as a researcher. While on the way to the hospital he lost consciousness. Upon arrival, the patient was noted to have a petechial rash, was suspected of having meningococcal disease, and was treated with ceftriaxone. He later had a respiratory arrest. Attempted resuscitation was unsuccessful, and he was declared dead approximately 3 hours after his arrival.

On the day of the patient’s death, hospital A notified the local health department and CDPH of the case of suspected meningococcal disease. On April 29, hospital A notified OSHA, which notified CalOSHA that the deceased had worked in a laboratory conducting *N. meningitidis* vaccine research. Hospital A evaluated potentially exposed emergency department staff members and research laboratory employees; all persons found to have been exposed were immediately assessed for symptoms of meningococcal disease and offered postexposure chemoprophylaxis. Laboratory A voluntarily closed on April 30. No additional cases of meningococcal disease were identified among emergency department or laboratory staff members. The local health department identified other close contacts of the patient and ensured that they received postexposure chemoprophylaxis.

Blood and tissue specimens from the patient were sent to the CDPH Microbial Diseases Laboratory for isolation and serogroup identification. *N. meningitidis* serogroup B was identified in the clinical specimens by polymerase chain reaction. The patient had worked with *N. meningitidis* serogroup B isolates in the weeks and days before his death.

## Investigation Findings

CalOSHA, OSHA, and CDPH initiated an investigation. Laboratory A was inspected, and employees were interviewed about their training as well as laboratory practices and protocols and were asked to demonstrate how procedures were performed. Multiple breaches in recommended laboratory safety practices were identified (Tables 1 and 2), including manipulation of *N. meningitidis* isolates on an open laboratory bench (2,5). The inspection team made recommendations for safe handling of *N. meningitidis* isolates and use of appropriate personal protective equipment. Laboratory A microbiologists working with *N. meningitidis* isolates had not been offered quadrivalent meningococcal vaccine, as recommended by ACIP (4). At the conclusion of the investigation, OSHA issued three citations classified as serious for failure to protect laboratory workers.

### Discussion

Although occupationally acquired meningococcal disease is rare, it is a known risk among microbiologists who work with *N. meningitidis* isolates (6–8). Investigations of laboratory-acquired cases of meningococcal disease in the United States have demonstrated a many-fold higher attack rate for microbiologists compared with the U.S. general population aged 30–59 years and a case fatality rate of 50%, more than triple the 12%–15% case fatality rate associated with disease in the general population (9). In almost all cases, infected microbiologists had manipulated sterile-site isolates on an open laboratory bench outside of a biosafety cabinet (2,6). Manipulating *N. meningitidis* isolates outside a biosafety cabinet is known to be associated with a high risk for contracting meningococcal disease (7).

To decrease the risk of transmission to laboratory workers handling invasive *N. meningitidis* strains (serogroups A, B, C, Y, and W), CDC recommends the use of enhanced biosafety level two (BSL-2) containment practices, where BSL-2 requirements are met and some BSL-3 practices also are adopted (2). Updated recommendations for microbiologists manipulating *N. meningitidis* strains were published in January 2012 as a supplement to the *Biosafety in Microbiological and Biomedical Laboratories* guide and include the use of a nonrecirculating biosafety cabinet and the following personal protective equipment: disposable closed front laboratory coat, double gloves, fit-tested N95 filtering facepiece or higher level respiratory protection, and eye protection (2,5). In California, personnel using respirators also must be enrolled in a respiratory protection program (10).

What is already known on this topic?

Working with *Neisseria meningitidis* isolates without adequate protection on the open laboratory bench can result in aerosol transmission of the bacteria. Meningococcal disease is severe and can be fatal. Among laboratory-acquired meningococcal disease cases, the case fatality rate was 50% in one study, significantly higher than the case fatality rate in the general population. The Advisory Committee on Immunization Practices (ACIP) has published immunization guidelines for laboratory workers who are routinely exposed to isolates of *N. meningitidis*.

What is added by this report?

A laboratory researcher who worked with *N. meningitidis* died from serogroup B meningococcal disease. An investigation identified deficiencies in training and practices in laboratory A, including manipulating cultures outside of a biosafety cabinet. Additionally, laboratory workers who routinely worked with *N. meningitidis* had not been vaccinated in accordance with current ACIP recommendations.

What are the implications for public health practice?

Adequate safety training for laboratory personnel, adherence to recommendations for safe handling of *N. meningitidis* isolates, and vaccination (where indicated) are necessary to reduce the risk for disease among laboratory workers.

Although this fatal case of serogroup B meningococcal disease was not vaccine-preventable by meningococcal vaccines currently licensed in the United States, licensed vaccines to protect against serogroup A, C, Y, and W-135 disease are available. ACIP recommends meningococcal vaccination for microbiologists who are routinely exposed to isolates of *N. meningitidis* (3,4). The CalOSHA Aerosol Transmissible Diseases Standard also requires that California employers offer all vaccinations as recommended by applicable public health guidelines for specific laboratory operations (1,4). A serogroup B vaccine (Bexsero, Novartis) was licensed in Europe, Australia, and Canada in 2013 and has received a “breakthrough therapy” designation from the Food and Drug Administration.

Employers should be familiar with laboratory biosafety recommendations and ensure that a laboratory biosafety program is in place. Employers also should ensure that laboratory staff are trained, adhere to recommended biosafety practices and procedures, and are offered recommended vaccines.

## Figures and Tables

**TABLE 1:** Selected breaches in recommended laboratory practices for *Neisseria meningitidis* that were observed by an inspection team after the death of a laboratory worker — California, 2012

Activity	Observed practice	Recommended practice
Flaming of Gram stain slide	Slide not allowed to completely air dry before flaming. This activity was conducted on the open bench.	Allow the slide to air dry before applying fixation. Use alternative methods (e.g., alcohol fixation) in the BSC.
Plate spreading	A disposable plate spreader was used to saturate the plate with the organism. The activity was conducted on the open bench.	A cotton-tipped swab could be used instead of a plastic spreader to reduce the amount of generated aerosol. If plate spreading is necessary, it should be conducted in the BSC.
Plate scraping	A disposable plastic plate scraper was used to harvest the bacteria on the plate. This activity was conducted on the open bench.	Plate scraping is not recommended, but if necessary should be performed in the BSC with appropriate PPE.
Flaming loops	Transfer loops used to inoculate media were flamed on the open bench.	Open flames are no longer universally recommended. Electric furnaces are an alternative. Disposable transfer loops used in the BSC are preferable.
Re-suspension of solution	A solution containing substantial concentrations of viable organism was inoculated with an inactivating enzyme. The solution was vigorously pipetted to create a homogenous solution. This activity occurred 10 minutes into the enzymatic reaction.	This activity should be performed in the BSC. Manufacturer recommends a 20–30 minute treatment time for the enzymatic reaction.
Opening discard bin	The biohazard discard bin lid was foot-pedal operated and opening can rapidly generate an aerosol.	Infectious material should be manipulated in the BSC. Discards should be disposed of in a biohazard bag in the BSC. Biohazard bags should be sealed and wiped down before they are transferred to the biohazard bin outside the BSC.
Discarding plate scraper and spreader	Microbiologists dropped contaminated scrapers and spreaders into an open discard bin located on the floor after working with them on the open bench, potentially generating aerosols.	Spreaders and scrapers should only be used in the BSC. Contaminated spreaders and scrapers should be placed in either a discard pan or biohazard bag. The bag or container should be sealed or covered with a lid and wiped down before removal from the BSC.

**Abbreviations:** BSC = biological safety cabinet; PPE = personal protective equipment.

**TABLE 2:** Selected breaches in recommended personnel protective equipment practices for *Neisseria meningitidis* that were observed by an inspection team after the death of a laboratory worker — California, 2012

Personal protective equipment	Observed practice	Recommended practice
Laboratory coat	Cloth laboratory coats were worn. Coats were not routinely decontaminated.	Disposable closed front laboratory coats are preferred. If reusable coats are used, they should be routinely decontaminated and then laundered.
Gloves	Microbiologists wore a single pair of latex gloves while working on the open bench and in the BSC.	BSL-2+ practices warrant using double gloves. The outer gloves should be removed and placed in the biohazard bag or pan in the BSC. Then inner gloves can be removed outside the BSC. Microbiologists should immediately wash their hands upon removing inner gloves.
Eye protection	Microbiologists wore their regular prescription eye glasses for eye protection.	Regular eye glasses are not considered eye protection. Wrap-around eye protection, goggles, or face shields are preferred.
Respiratory protection	Laboratory staff only wore N95 respirators while cleaning up spills.	BSL-2+ practices warrant the use of a respirator that is at least as protective as a fit-tested NIOSH-certified N95 filtering facepiece respirator particularly when culturing large volumes of *N. meningitidis*.

**Abbreviations:** BSC = biological safety cabinet; BSL = biosafety level; NIOSH = National Institute for Occupational Safety and Health.
